# Exploring Differences in the Lives and Well-Being of Sexual and Gender Minority Adults Experiencing Homelessness Relative to Their Cisgender Heterosexual Counterparts

**DOI:** 10.1089/heq.2018.0068

**Published:** 2019-03-14

**Authors:** Vanessa Schick, Laura Witte, Lourence Misedah, Wendy Benedict, Kinnon Falk, Carlie Brown, Frances Isbell

**Affiliations:** ^1^ Department of Management, Policy and Community Health, School of Public Health, University of Texas Health Science Center at Houston (UTHealth), Houston, Texas.; ^2^ Healthcare for the Homeless Houston, Houston, Texas.

**Keywords:** bisexual, gay, health equity, homeless, homelessness, jail, lesbian, LGBTQ, transgender

## Abstract

**Purpose:** To better understand the lives and experiences of sexual and gender minority (SGM) adults experiencing homelessness relative to their cisgender heterosexual (non-SGM) counterparts.

**Methods:** A modified time-location sampling strategy was used to reach a diverse sample of individuals with experiences of homelessness. Interviewer or self-administered paper-based surveys were administered to participants on location.

**Results:** SGM and non-SGM participants reported significant differences in the age at which they became homeless, their current housing, and experiences of violence over the past year. SGM participants reported poorer mental health than their non-SGM counterparts.

**Conclusion:** SGM adults may be uniquely impacted by their experiences of homelessness.

## Introduction

The disproportionate number of homeless youth who identify as sexual and gender minorities (SGM) is widely cited, with considerable research and resources directed toward rectifying this inequity.^1–4^ Elevated rates of homelessness among SGM youth is often attributed to familial rejection of the youths' sexual or gender identity.^1^ What remains unknown is whether homelessness among SGM youth has only recently increased or whether this disparity has gone undocumented over time. Although either is conceivable, societal acceptance of SGM persons has increased over the years,^5^ suggesting that SGM youth of earlier generations may have been historically overrepresented among homeless youth. If this is the case, SGM homeless youth of earlier generations may have since transitioned into a disproportionate number of housing unstable SGM adults. Yet, there is much less known about SGM adults experiencing homelessness, with only one known study comparing the causes of homelessness between SGM adults and their cisgender heterosexual (non-SGM) counterparts.^6^ Although informative, the study was not designed to assess SGM-specific reasons for homelessness and found no differences between SGM and non-SGM adults.^6^ A recent review on SGM adults experiencing homelessness specifically pointed to the lack of information on the pathways to homelessness as a critical gap in the literature.^7^

In addition to a lack of understanding about the pathways to homelessness among SGM adults, the current literature tends to focus on HIV or substance use,^8,9^ with little information about the ways in which the lived experiences of SGM adults experiencing homelessness may differ from their non-SGM counterparts. The sampling methods used in previous studies are similarly limited with the majority sampling homeless individuals from within SGM communities.^10–13^ Instead, assessing SGM identity among a sample of individuals experiencing homelessness would increase generalizability and allow for comparisons between SGM and non-SGM adults. To partially address this gap, the present study explores differences in causes, experiences, and health of a diverse sample of SGM and non-SGM adults experiencing homelessness.

## Methods

The present study was conducted as part of a need assessment for a Federally Qualified Health Center that provides care exclusively to individuals with experiences of homelessness. Most research with individuals experiencing homelessness does not include a representative sample given the sampling strategy challenges with this population. To ensure that our sample included individuals with diverse experiences of homelessness, a modified time-location sampling (TLS) strategy was used in the present study. TLS has been used previously to sample hard-to-reach populations often in the context of infectious disease surveillance.^14,15^ TLS involves the identification of a complete set of venues and the days and times at which the population visits these venues. The venues in this study included organizations that serve the homeless community, permanent supportive housing sites that house individuals who have experienced homelessness, and outdoor locations where people experiencing homelessness congregate. A universe of all possible venue, day-time units (VDTs) was established, and VDTs were drawn randomly to make a calendar of visits to venues for data collection. Teams of at least three people visited venues in 4-h blocks to count and approach all individuals at the venue with an invitation to participate in the study. Interviewer or self-administered paper-based surveys were administered to individuals on location, and participants were provided with a hygiene kit at completion of the program.

Survey questions included sex at birth,^16^ gender identity,^16^ sexual identity,^16^ race/ethnicity,^17^ education,^18^ employment,^19^ felony history, and veteran status.^20^ Participants were asked where they slept in the past 12 months, the place they spent their past night, and perceived causes for homelessness.^21^ Participants were asked to indicate needed services from a list of local services (e.g., clothing, ID). Consistent with the CDC's Health-Related Quality of Life measure and the mental health item in the Homeless Management Information System, participants rated their mental and physical health on a scale from poor to excellent.^22,23^ Procedures were reviewed by a consumer advisory board before implementation. Secondary data analysis was approved by the UTHealth IRB. Differences between SGM and non-SGM participants were explored using chi-square, *t*-tests, logistic regression, and analysis of covariance.

## Results

SGM participants were, on average, a decade (*M*=9.70, SD=1.14) younger (*t*=4.87, *p*<0.001) than non-SGM participants (Table 1). In the past 12 months, after controlling for age and race, SGM participants were more likely to spend a night in jail or on the streets (Table 2). When asked where they slept the night before and where they most frequently slept in the past 30 days, the largest percentage of non-SGM reported that they slept in permanent supportive housing (24.7%, *N*=54), whereas the largest percentage of SGM participants reported sleeping on the streets (33.3%, *N*=11).

**Table 1.** Participant Characteristics (*N*=423)

Sample characteristics	Total sample	SGM	Non-SGM	*p*
	% (*N*)	% (*N*)	% (*N*)	
Gender				
Cis man	65.48 (277)	54.90 (28)	66.94 (249)	
Cis woman	32.39 (137)	27.45 (14)	33.06 (123)	
Trans woman	1.70 (7)	13.73 (7)	—	
Trans man	0.43 (2)	3.92 (2)	—	
Sexual identity				
Lesbian or gay	4.19 (18)	36.00 (18)	—	
Straight	88.14 (379)	10.00 (5)	100.00 (372)	
Bisexual	5.81 (25)	50.00 (25)	—	
Pansexual	0.47 (2)	4.00 (2)	—	
Other (none, unsure, etc.)	1.40 (6)	—	—	
Age at entry, years				
18–19	2.80 (12)	8.16 (4)	2.28 (8)	27.81^***^
20–29	10.75 (46)	18.37 (9)	9.69 (34)	
30–39	16.82 (72)	28.57 (14)	15.38 (54)	
40–49	14.25 (61)	22.45 (11)	13.39 (47)	
50–59	38.08 (163)	22.45 (11)	39.60 (139)	
60–69	16.59 (71)		18.80 (66)	
70+	0.70 (3)		0.85 (3)	
Race				
White	30.04 (134)	44.00 (22)	29.81 (110)	4.11^*^
Black/African American	62.33 (278)	48.00 (24)	63.69 (235)	4.59^*^
American Indian/Native American	2.47 (11)	2.00 (1)	2.44 (9)	
Asian	0.90 (4)	0.00 (0)	1.08 (4)	
Native Hawaiian/Pacific Islander	0.90 (4)	0.00 (0)	0.54 (2)	
Other	7.85 (35)	10.00 (5)	6.50 (24)	
Ethnicity				
Not Hispanic/Latino(a)	88.89 (376)	91.49 (43)	88.45 (314)	
Hispanic/Latino(a)	11.11 (47)	8.51 (4)	11.55 (41)	
Education				
Less than high school	23.09 (103)	26.00 (13)	24.32 (90)	
High School/GED	42.38 (189)	48.00 (24)	40.27 (149)	
Vocational or some college	25.78 (115)	18.00 (9)	27.03 (100)	
College degree or higher	8.74 (39)	8.00 (4)	8.38 (31)	
Employment				
Full time work	7.98 (36)	11.76 (6)	8.09 (30)	
Part-time work	7.76 (35)	7.84 (4)	8.36 (31)	
Day labor/labor pool	2.00 (9)	1.96 (1)	2.16 (8)	
Unemployed	38.80 (175)	39.22 (20)	39.89 (148)	
Disabled	27.72 (125)	35.29 (18)	26.95 (100)	
Other	15.74 (71)	3.92 (2)	14.56 (54)	
Veteran status				
Never served in the military	89.03 (357)	93.02 (40)	88.44 (306)	
I am a U.S. Veteran	10.47 (42)	6.98 (3)	11.27 (39)	
Now on active duty	0.50 (2)	0.00 (0)	0.29 (1)	
Felony status				
No felony conviction	58.70 (216)	47.60 (20)	50.1 (196)	
Convicted of felony	41.30 (152)	52.40 (22)	39.9 (130)	

^*^ *p*<0.05, ^***^p<0.001. Only age was significant after adjusting for the False Discovery Rate using the Benjamini-Hochberg prodecure.

SGM, sexual and gender minority.

**Table 2.** Places Slept in the Past 12 Months by Sexual and Gender Identity

Spent a night in past 12 months	Total sample	SGM	Non-SGM	AOR	*p*
	% (*N*)	% (*N*)	% (*N*)		
Rent or own home	24.32 (71)	24.24 (8)	24.32 (63)	1.03	
Car or other vehicle	19.93 (60)	29.41 (10)	18.73 (50)	1.47	
Detox	9.70 (29)	11.43 (4)	9.47 (25)	1.02	
Family/friends	38.92 (123)	46.15 (18)	37.91 (105)	0.86	
Hospital	28.06 (87)	35.14 (13)	27.11 (74)	1.67	
Jail	22.15 (68)	45.71 (16)	19.12 (52)	3.21	^***^
Motel	33.01 (102)	44.44 (16)	31.50 (86)	1.33	
Transitional housing	41.41 (135)	37.84 (14)	41.87 (121)	0.70	
Permanent supportive housing	35.63 (119)	21.62 (8)	37.37 (111)	0.54	
Emergency shelter	55.87 (200)	61.90 (26)	55.06 (174)	1.12	
Streets	51.38 (167)	76.92 (30)	47.90 (137)	3.90	^****^
Residential treatment	24.13 (76)	31.58 (12)	23.10 (64)	1.36	
Other	18.70 (23)	41.67 (5)	16.22 (18)	2.40	

AOR=Odds ratio adjusted for race and age. ^***^p<0.005, ^****^p<0.001. Relationship to spending a night in the jail or the streets was significant after adjusting for the False Discovery Rate using the Benjamini-Hochberg procedure.

SGM participants reported becoming homeless at a younger age (*M*=32.32, SD=12.58) than their non-SGM counterparts (*M*=38.88, SD=14.98, *t*=4.87, *p*<0.001). SGM and non-SGM participants did not differ in the amount of time they spent homeless. The most common reason for homelessness reported by participants was that they lost their job or family income (non-SGM: 53.3%, *N*=178; SGM=52.2%, *N*=24). SGM participants were more likely to report that they lost housing due to a change in assistance (e.g., temporary assistance for needy families) eligibility (SGM: 28.3%, *N*=13, *χ*^2^=4.33, *p*<0.05) or because of their sexuality or gender identity (SGM: 17.4%, *N*=8, *χ*^2^=27.71, *p*<0.001).

Investigating experiences over the past year, SGM participants were twice as likely to report that they were attacked with a gun or knife (SGM: 32.6%, *N*=14, non-SGM: 15.5%, *N*=50; *χ*^2^=7.67, *p*<0.01) or forced or made to engage in sexual contact (SGM: 11.9%, *N*=5, non-SGM: 4.6%, *N*=15, *χ*^2^=3.81, *p*<0.05). SGM participants were also more likely to report transactional sex in the past year (SGM: 16.3%, *N*=7, non-SGM: 5.9%, *N*=19, *χ*^2^=6.18, *p*<0.05). These relationships became nonsignificant when entered into a multivariate model with sociodemographic characteristics.

When asked about current needs, permanent housing was the most common need for SGM and non-SGM participants. SGM participants were more likely to report that they needed assistance getting an ID (*χ*^2^=4.82, *p*<0.05), food stamps (*χ*^2^=5.74, *p*<0.05), food (*χ*^2^=6.05, *p*<0.05), and substance use treatment (*χ*^2^=6.47, *p*<0.05). Needs did not significantly differ between SGM and non-SGM participants after controlling for sociodemographic and living characteristics.

SGM and non-SGM participants did not significantly differ in self-reported physical health. SGM participants reported significantly poorer mental health relative to their non-SGM counterparts (Table 3) in bivariate and multivariate models controlling for sociodemographic and living characteristics (*F*=6.73, *p*<0.01).

**Table 3.** SGM Identity to Self-Reported Mental Health

Variables	*F*	η_*p*^2^_
Age	3.78	0.02
Age first homeless	0.16	0.00
Night on street	0.32	0.00
Night in jail	1.21	0.01
Race: White	0.20	0.00
Race: Black	0.02	0.00
SGM	6.73^**^	0.03

^**^ *p*<0.01

## Discussion

This is the first known study to compare the lives, experiences, and needs of SGM and non-SGM adults experiencing homelessness. Consistent with previous research,^6^ SGM and non-SGM participants reported few differences in their reasons for homelessness with the majority of participants in both groups reporting that their first experience of homelessness was as an adult. A sizable minority of SGM participants reported that they had to leave previous residences for sexuality or gender-related reasons. One gay-identified cisgender man reported that his homelessness was due to discrimination he experienced when landlords would not include his husband on the lease. Future researchers should continue to explore the complex and subtle ways in which one's sexuality and gender identity may increase their vulnerability for homelessness (e.g., employment discrimination and partner benefits) not captured in the present study.

In the present study, SGM individuals experiencing homelessness were more likely to report recent gun/knife violence, sexual assault, and elevated service needs, a difference that may be largely attributable to differences in housing. SGM participants were more likely than non-SGM participants to report having spent a night on the streets or jail with the largest percentage of SGM participants reporting that they slept on the streets over the past year. The driving mechanism behind this housing difference remains unknown. It may be that SGM individuals are less likely to seek housing; that those who are in housing/shelters are less likely to identify as SGM; or that people who identify as SGM are not universally welcome in privately funded shelters/housing.^13,24^ One participant interviewed in religious housing indicated that she used to identify as a lesbian, but now identifies as heterosexual/straight for religious/moral reasons. Perhaps relatedly, non-SGM and SGM participants significantly differed in their self-reported mental health. Together, these findings suggest that SGM adults experiencing homelessness may benefit from the intentionally-inclusive housing and service strategies designed for SGM youth experiencing homelessness.^13,24^

The present study has several limitations, including the sample size, which limited both generalizability and our ability to disaggregate SGM participants to explore the diversity of their experiences.^2^ Despite limitations, differences between SGM and non-SGM participants experiencing homelessness may have important policy and practice implications. For instance, adding sexual orientation and gender identity to the Fair Housing Act could increase access to housing and perceived security while housed for SGM individuals. The current lack of housing protection combined with the adverse experiences reported by SGM participants in the present study supports the potential benefit of assessing SGM identity on standardized assessment tools used to determine housing prioritization. Further consideration and development of tailored evidence-based interventions and system-level strategies are needed to reduce sexuality and gender-based barriers to health and housing for SGM individuals experiencing homelessness.

## Abbreviations Used

HMIS: Homeless Management Information System
SGM: sexual and gender minority
TLS: time-location sampling
VDTs: venue, day-time units

## References

[1] Durso LE, Gates GJ Serving our Youth: Findings from a National Survey of Services Providers Working with Lesbian, Gay, Bisexual and Transgender Youth who are Homeless or at Risk of Becoming Homeless. Los Angeles: The Williams Institute with True Colors Fund and The Palette Fund, 2012

[2] Keuroghlian AS, Shtasel D, Bassuk EL Out on the street: a public health and policy agenda for lesbian, gay, bisexual, and transgender youth who are homeless. Am J Orthopsychiatry. 2014;84:66 24826829 10.1037/h0098852 PMC4098056

[3] Edidin JP, Ganim Z, Hunter SJ, et al. The mental and physical health of homeless youth: a literature review. Child Psychiatry Hum Dev. 2012;43:354–375 22120422 10.1007/s10578-011-0270-1

[4] Medlow S, Klineberg E, Steinbeck K The health diagnoses of homeless adolescents: a systematic review of the literature. J Adolesc. 2014;37:531–542 24931556 10.1016/j.adolescence.2014.04.003

[5] Glick SN, Cleary SD, Golden MR Brief report: increasing acceptance of homosexuality in the United States across racial and ethnic subgroups. JAIDS J Acquired Immune Defic Syndromes. 2015;70:319–322 10.1097/QAI.0000000000000740 26381102

[6] Flentje A, Leon A, Carrico A, et al. Mental and physical health among homeless sexual and gender minorities in a major urban US city. J Urban Health. 2016;93:997–1009 27699581 10.1007/s11524-016-0084-3 PMC5126021

[7] Ecker J, Aubry T, Sylvestre J A review of the literature on LGBTQ adults who experience homelessness. J Homosex. 2019;66:297–323 10.1080/00918369.2017.1413277 29206576

[8] Flentje A, Shumway M, Wong LH, et al. Psychiatric risk in unstably housed sexual minority women: relationship between sexual and racial minority status and human immunodeficiency virus and psychiatric diagnoses. Womens Health Issues. 2017;27:294–301 28108194 10.1016/j.whi.2016.12.005 PMC5435529

[9] Salem BE, Nyamathi A, Reback C, et al. Unmet physical and mental healthcare needs among stimulant-using gay and bisexual homeless men. Issues Ment Health Nurs. 2015;36:685–692 26440871 10.3109/01612840.2015.1021938 PMC4801108

[10] Cahill S, Trieweiler S, Guidry J, et al. High rates of access to health care, disclosure of sexuality and gender identity to providers among house and ball community members in New York City. J Homosex. 2018;65:600–614 28537845 10.1080/00918369.2017.1328221

[11] Krause KD, Kapadia F, Ompad DC, et al. Early life psychosocial stressors and housing instability among young sexual minority men: the P18 cohort study. J Urban Health. 2016;93:511–525 27169631 10.1007/s11524-016-0049-6 PMC4899333

[12] Nyamathi A, Reback CJ, Salem BE, et al. Correlates of self-reported incarceration among homeless gay and bisexual stimulant-using young adults. West J Nurs Res. 2015;37:799–811 24733231 10.1177/0193945914530521 PMC4197114

[13] Schmitz RM, Tyler KA LGBTQ young adults on the street and on campus: identity as a product of social context. J Homosex. 2018;65:197–223 28402223 10.1080/00918369.2017.1314162

[14] MacKellar DA, Gallagher KM, Finlayson T, et al. Surveillance of HIV risk and prevention behaviors of men who have sex with men—a national application of venue-based, time-space sampling. Public Health Rep. 2007;122:39–47 17354526 10.1177/00333549071220S107 PMC1804106

[15] Zhao J, Cai R, Chen L, et al. A comparison between respondent-driven sampling and time-location sampling among men who have sex with men in Shenzhen, China. Arch Sex Behav. 2015;44:2055–2065 25239658 10.1007/s10508-014-0350-y

[16] Cahill S, Singal R, Grasso C, et al. Do ask, do tell: high levels of acceptability by patients of routine collection of sexual orientation and gender identity data in four diverse American community health centers. PLoS One. 2014;9:e107104 25198577 10.1371/journal.pone.0107104 PMC4157837

[17] Data collection standards for race, ethnicity, sex, primary language, and disability status. 2018. U.S. Department of Health and Human Services. Available at https://minorityhealth.hhs.gov/omh/browse.aspx?lvl=3&lvlid=53 Accessed 32, 2019

[18] Centers for Disease Control and Prevention. 2017. Behavioral risk factor surveillance system. Available at https://www.cdc.gov/brfss/questionnaires/pdf-ques/2017_BRFSS_Pub_Ques_508_tagged.pdf Accessed 32, 2019

[19] Ridolfo H. Testing of the National HIV Behavioral Surveillance System: Results of Interviews Conducted 1/13/2011–4/5/2011. Hyattsville, MD: National Center for Health Statics, 2011

[20] Holder K, Raglin D. Evaluation of the revised veteran status question in the 2013 American community survey, 2014

[21] Troisi CL, D'Andrea R, Grier G, et al. Enhanced methodologies to enumerate persons experiencing homelessness in a large urban area. Eval Rev. 2015;39:480–500 26468235 10.1177/0193841X15610191

[22] Centers for Disease Control and Prevention. Measuring Healthy Days: Population Assessment of Health-Related Quality of Life. Atlanta, GA CDC, 2000

[23] U.S. Department of Housing and Urban Development. HMIS Data Standards Manual. Washington, D.C.: HUD Exchange, 2017

[24] Mottet L, Ohle J Transitioning our shelters: making homeless shelters safe for transgender people. J Poverty. 2006;10:77–101

